# The dynamics of the immune system and gut in the pathophysiology of multiple sclerosis

**DOI:** 10.1016/j.ebiom.2022.104273

**Published:** 2022-09-14

**Authors:** eBioMedicine

Multiple sclerosis is an autoimmune disease involving the CNS. The typical hallmarks of multiple sclerosis are inflammation and demyelination, coupled with gliosis and neurodegeneration. Multiple sclerosis affects an estimated 2·8 million people globally and places a substantial burden on individuals, families, society, and health-care systems. Since 2010, there have been large advances in the diagnosis, treatment, and understanding of multiple sclerosis pathophysiology. Disease modifying therapies have been life altering in one type of multiple sclerosis, refractory-remitting multiple sclerosis. However, there are currently no highly efficacious therapies against the other, progressive forms of multiple sclerosis. Approximately two-thirds of people with multiple sclerosis continue to experience disparities in care as they live in countries where there are no national guidelines for the diagnosis and treatment of their condition. An understanding of the emerging and established players in multiple sclerosis pathophysiology, such as the gut and immune system, underpins the quest to find new therapies.

Multiple sclerosis manifests clinically in two forms: relapsing and progressive. The former is characterised by episodes of neurological dysfunction, followed by complete, partial, or no remission. Progressive multiple sclerosis is further sub-divided into primary progressive and secondary progressive multiple sclerosis. Fewer than 10% of patients have primary progressive multiple sclerosis at disease onset. Irrespective of the clinical course of the disease, diagnosis is typically between 30 and 40 years of age, and women are twice as likely to be affected compared with men. The discovery that B cells are pivotal in immune dysfunctionality has led to the development of anti-CD-20 depletion therapies, such as ocrelizumab and ofatumumab. An observational study that aimed to establish the safety and clinical outcomes of ocrelizumab in a community-based cohort in the USA reported that ocrelizumab was effective in controlling relapse risk. In the study, only 12 ·1% of people with multiple sclerosis discontinued use with ocrelizumab because of clinical progression or relapse. However, this study found that infections resulting in hospitalisation were a concern, especially in individuals who were older or who had disabilities.

Despite the progress made, the global prevalence of multiple sclerosis continues to increase. As the genetic risk of developing multiple sclerosis has not changed, it is speculated that this increase is due to environmental factors that have been implicated in the disease, such as obesity, smoking, and vitamin D deficiency. New insights have emerged in the inherited genetic risk of developing multiple sclerosis. A study in *eBioMedicine* published in May showed a significant differential expression of mature miRNAs and isomiRs from hsa-mir-26a-2 and hsa-mir-199a-1 in B cells from sub-groups of people with refractory-remitting multiple sclerosis and healthy controls. This study showed that multiple sclerosis-associated single nucleotide polymorphisms in sequence determinants of primary-miRNA processing can affect the expression of mature miRNAs and implicates these variants as a risk factor for multiple sclerosis. A landmark study in *Science* implicated Epstein-Barr virus (EBV) infection as a risk factor for multiple sclerosis. The study showed in a cohort of 10 million young adults actively serving in the US military that multiple sclerosis increased 32-fold after infection with EBV, but not with similarly transmitted viruses, such as cytomegalovirus. Expression of neurofilament light chain, a marker of neuroaxonal degeneration, was increased only after EBV seroconversion.

Another important emerging player in multiple sclerosis is the gut microbiome, although its role in multiple sclerosis is not as well studied as it is in other CNS disorders. A clinical trial published in *PNAS* analysed the gut microbiomes of 71 people with refractory-remitting multiple sclerosis and 71 healthy controls showed that *Akkermansia muciniphila* and *Acinetobacter calcoaceticus* were increased in people with multiple sclerosis which *in vitro* led to the skewing of the immune system towards a pro-inflammatory phenotype with attenuated regulatory T cell (Treg) activity. This finding was reinforced in a small correlative study in *Journal of The Neurological Sciences* with 17 children with refractory-remitting multiple sclerosis that were followed-up for a mean duration of 19·8 months. Increased levels of *Fusobacteria* were associated with increased relapse risk. Another study in *PNAS* used microbial composition data coupled with metagenomic functional and metabolite data to ascertain the alterations of the ecological and functional gut microenvironment in different stages of multiple sclerosis. People with refractory-remitting multiple sclerosis had reduced levels of microbial butyrate and propionate biosynthesis in the gut, whereas people with secondary progressive multiple sclerosis had an enhancement of microbial DNA mismatch repair and excessive faecal oxidation. This study facilitates the notion that microbiome data assisted management of multiple sclerosis could prevent disease progression.

The interaction between microbial components and various clinical and laboratory indices in multiple sclerosis is not well understood. A study in *eBioMedicine* by Cantoni and colleagues addressed this question by conducting a series of elegant multiomics analyses on data from the gut microbiome, peripheral immune cells, serum metabolites, and dietary contents. This study was done in 24 people with refractory-remitting multiple sclerosis and 25 healthy controls that were matched for age and sex. A significant correlation was found in people with multiple sclerosis, linking increased meat intake with decreased gut microbe *B thetaiotaomicron*, increased peripheral T helper cells (Th17), and an increased abundance of meat-associated blood metabolites. The microbiome and metabolome profiles of the people with multiple sclerosis and controls remained stable over 6 months. Although this study was small, single-centre, and people with multiple sclerosis were not stratified into stages, it provides intriguing evidence that diet affects the pathophysiology of multiple sclerosis.

Further evidence is accumulating about the modulation of the immune response in multiple sclerosis by external factors, such as diet, calorie restriction, and age. A study in *Cell* provided evidence to show that there were reduced propionic acid (PA) amounts in the serum and stool of people with multiple sclerosis compared with healthy controls. PA is present in dairy products, such as butter and cheese, and in humans is produced from the breakdown of amino acids and oxidation of fatty acids. PA supplementation in patients with multiple sclerosis who were receiving immunotherapy, as well as in patients who were not taking therapy, restored the Th17 and Treg imbalance after 2 weeks by increasing expression of Treg-cell-inducing genes in the intestine. Treg morphology, induction, and mitochondrial functionality were restored. These changes resulted in a less frequent annual relapse and disability stabilisation after 1 year, and reduced brain atrophy over a 3-year supplementation period. Fitzgerald and colleagues published in *eBioMedicine* that intermittent energy restriction (ER) also modulates the immune system in multiple sclerosis. A total of 36 people with multiple sclerosis were randomly assigned to three groups: control diet, daily ER, or intermittent ER. In the 86% of people with MS that completed the study, the intermittent ER group showed a significant reduction in effector memory and Th1 cell subsets with a proportional increase in naïve subsets. This result was not observed in the group receiving daily ER. Metabolome analysis showed an increase in acyl carnitine metabolism and changes in phosphatidylethanolamine and plasmalogens, which were more significant in the intermittent ER group than in the group receiving daily ER. A study in *eBioMedicine* published in July showed that although numerous aspects of T-cell ageing are conserved in multiple sclerosis, people with multiple sclerosis who are older than 60 years have increased levels of CD4 T cells, particularly activated ones. This phenomenon is coupled with a significant decrease of CTLA-4 on both CD4 (p=0·014) and CD8 T cells (p=0·009) and an increased B cell co-stimulatory expression.

All the aforementioned studies are either small or proof-of-concept studies, but indicate important avenues for further research. Large, well conducted studies and clinical trials with robust endpoints involving patients with different multiple sclerosis disease severities are required to further elucidate the mechanistic functions and interplay between the gut and immune system and the modulation of the latter. These studies will lead to the ultimate goal of personalised, evidence-based management and therapy for multiple sclerosis. *eBioMedicine,* as a translational platform, seeks to publish work that will facilitate the realisation of this goal.

